# Cohort Differences and Similarities in Women's Attitudes About Self and Aging

**DOI:** 10.1093/geroni/igab046.211

**Published:** 2021-12-17

**Authors:** Aurora Sherman, Jamila Bookwala

## Abstract

This panel focuses on four complementing and international views of women’s aging, with a special emphasis on cohort comparisons and using three different studies of women, with contrasting methodological frameworks. In so doing, we present evidence related to trends in social percepetions of aging, attitudes about aging and identity, and ideas about control and objectification. Dr. Newton presents data on older Canadian women showing the connection between physical aging and identity maintenance, using both qualitative and quantitative data and using the lifecourse perspective. Dr. Ryan, using data from the Health and Retirement Study to compare cohorts of women from the 2008 and 2018 HRS waves, reports cohort differences in negative self-perceptions of aging, and that both cohort and negative self-perfections are associated with life satisfaction, using the life course developmental framework. Ms. Tran compares younger and older cohorts of women on a measure of self-objectification, finding that the older cohort reported lower objectification, consistent with a selection, optimization, and compensation (SOC) model. Finally, Dr. Sherman, using the same data set as Ms. Tran, shows that control beliefs are associated with objectification, regardless of cohort, consistent with objectification theory predictions of consistency over time regarding the impact of objectification experiences. Dr. Jamila Bookwala will provide discussion of this group of papers.

